# Treatment of Acute Pleuritis

**Published:** 1892-09-10

**Authors:** 


### TREATMENT OF ACUTE PLEURITIS.

The treatment of acute pleurisy may be said to be simple; but, unfortunately, it is not very efficient. Of course, a great deal may be done, but we might well wish that much more could be done for the relief and cure of our patients suffering from this affection. The treatment of pleurisy formed the subject of an instructive paper by Dr. Rickards to the Midland Medical Society. He asked, "Can we by treatment prevent effusion in a pleurisy which, without treatment, would be effusive?" He said he thought not. "With this object in view, resort has been had to blistering, bleeding, poultices, purgatives, diuretics, diaphoretics, mercury, opium, aconite, quinine, and the more specific antipyretics; but, as far as my experience goes, without avail. I am ready to admit that in some cases of acute pleurisy, as in most inflammatory affections, a calomel purge at the onset does good by freeing the secretions and lowering arterial tension, and I have sometimes thought that in the early stage of acute pleurisy I have seen the severity and duration of the febrile stage modified by a few small doses of calomel and opium, but I have never been able to satisfy myself that any method of treatment has prevented effusion. Then comes the question, Have we any means of promoting the natural absorption of the effusion? Beyond keeping our patients in bed and improving the general health, I think not." Passing to the question of artificially removing the effused fluid, he said that we have four objects in view in undertaking this procedure, viz.: (1) The prevention or relief of urgent symptoms to prevent death from the effects of pressure of rapid and excessive effusion; (2) to shorten the duration of the disease; (3) to prevent a pleuritic effusion from becoming chronic; (4) to obviate the possibility of the collapsed lung. The author remarked that the question arises, When should we operate? To which he would answer, "Not until the febrile movement has ceased, unle that movement should continue more than a fortnight."

Dr. Castiaux, a Parisian practitioner, advocated in 1873 the removal of as much fluid as possible as soon as it was detected. He urged that the earlier and more complete its removal, the less likelihood there was of its recurrence; and more than that, he maintained that its removal extinguished the initial fever and ended the disease; but the author's experience does not enable him to go all the way with Castiaux. By operating in the earlier stage of the fever, he had found the pyrexia never extinguished and rarely reduced, and re-accumulation of fluid universal. He had no doubt in affirming that, when the febrile stage is over, the sooner the fluid is removed the less likely it is to re-accumulate, and the more rapid the recovery of the patient. A *resumé* of the treatment adopted by various Parisian physicians of eminence was given in the *Medical Press and Circular* for January 27th. and is of great interest. Thus, according to Germain Sér, the patient should be well alimented, in order to have strength to struggle against the invasion of the microbe. Thoracentesis should not be made before the twentieth or thirtieth day, otherwise the effusion will be sure to return. Professor Hayem, on the other hand, considers salicylate of sodium as capable, if given internally, of reducing considerably the amount of the liquid. He discards blisters. M. Lecorché follows the old classical method, as does also Professor Strauss. M. Talamon has confidence only in thoracentesis. However, he does not have recourse to it in every case, as many pleurisies get well by simple rest in bed. In any case he does not practice it before the third week. He orders no blisters, nor any diuretics, as he believes them hurtful. Professor Dieulafoy says that in acute pleurisy there are two indications—treatment of the pain and of the effusion. For the pain a small blister might be applied, or an injection of morphine given. When the liquid has exceeded a quart, tapping must be done, but all the liquid should not be drawn off at once, in order to avoid the accident so often witnessed in thoracentesis. The operation can be repeated a few days afterwards if necessary. The needle should be inserted in the eighth intercostal space, below the angle of the scapula. He never employs blisters, diuretics, or purgatives. M. Huchard submits his patient to a milk diet, and orders a mixture containing squills and digitalis, with an occasional blister. He considers that pleuritic patients in general are candidates for tuberculosis, and consequently should be looked after. M. Dujardin-Beaumetz is a warm partisan of revulsives. He employs large blisters, and renews them from time to time. M. Montard-Martin gives his preference to painting with tincture of iodine and the administration of quinine. M. Faisans said that when called at the *début* of acute pleurisy, he employs wet-cupping, in order to relieve the pain and the oppression. As long as the effusion remains within bounds he orders no other treatment; no blister as long as there is fever, no purgatives, no pilocarpine, and in general no diuretics, unless the patient insists on his "doing something." According to M. Faisans, there exists no medicinal treatment, properly speaking, for pleurisy. Thoracentesis is the treatment *par excellence* when the liquid shows no tendency to diminish.

We cannot omit further reference to the treatments with salicylates. Dr. H. Köster (*Ther. Monat.*, March, 1892) reports his trial of salicylic acid in the treatment of thirty-two cases of pleurisy, and thinks it is a remedy which deserves to be better known. Twenty-seven cases were of primary serous pleurisy, in which the lungs gave no sign of any change of a tubercular nature, and the exudate was a clear yellowish serum, and there was also no indication in the history of the possible presence of tuberculosis. The result was decidedly favourable in seventeen of these cases. Some of them were very ill, dulnes xisting throughout half of the chest, and the respiratory sound being in some entirely absent, in others intensely bronchial. Besides this, while some of the cases were new ones, the disease only having existed a few days, others were already of several weeks' standing. Most of the patients had fever when admitted, but in nearly every case this begun at once to diminish, and in a few days was entirely gone. Soon after beginning the use of the salicylic acid there began to be a resorption of the exudations, which advanced rapidly, and was usually complete in a short time. After two to four days there was a marked lessening of the exudate, and in most cases it had completely disappeared in from five to seven days, even when the exudations were very considerable. The dose was fifteen grains of salicylic acid, or salicylate of sodium in doses of twenty-two grains, three or four times a day. There were, on the whole, good results, although no constant action can be claimed for it. In some cases nothing was achieved by it, although they were seemingly of the same nature as those so favourably influenced by it.*

Previous to the above report, Dr. Déri had published in the *Pester Med. Chiv. Presse* a case of pleuritic effusion, in which salicylates were administered for rheumatism. Seen by Dr. Déri in the seventh week of the disease, he found his patient had severe bronchial catarrh and an effusion into the right pleura. After a week's treatment the catarrh decreased, the fever disappeared, and he then treated the effusion by iodides and diuretics, but without success. An operation was suggested, when the patient caught a chill, which set up acute rheumatism. The doctor administered salicylate of sodium, which produced considerable diaphoresis, and on the fifth day of the disease all rheumatic symptoms had disappeared, and on percussing the chest the respiration over the seat of the effusion was audible to a surprising degree. Since that

* *Ther. Gaz.*, May 16th, 1892.

time, the year 1885, Dr. Déri has prescribed salicylates in cases of pleuritic effusion with great success.*

Without attempting to deal fully with the question of treatment of acute pleurisy, we think that the above remarks will tend to illustrate our opening statements as to the present unsatisfactory means at our disposal, and that the salicylate of soda will do much towards obviating the necessity of repeated aspiration in many cases.

*Lancet, Oct. 10th, 1891.

